# Impact of problematic Khat use on mental health in Ethiopia’s Somali Regional State: a population study

**DOI:** 10.1136/bmjment-2025-302388

**Published:** 2026-07-28

**Authors:** Nasir Warfa, Roxanne C Keynejad, Elyas Abdullahi, Chris Willott, Ramadan Budel, Mahamed A Wali, Abdifetah A Shiek, Selamu Medhin, Mussie Abdosh Hassen, Abdulahi Hussen, Ahmed M Abdinasir, Abdikarim Mohamed M Abdi, Abdilahi E Mumin, Mohamud Salaad Mohamud, Solomon Teferra, Mustafa Al’Absi, Fadumo Osman, Iain Marshall, Charlotte Hanlon

**Affiliations:** 1 Institute of Health Science, Jigjiga University, Jijiga, Ethiopia; 2 Population Health Science, King’s College London Faculty of Life Sciences & Medicine, London, UK; 3 Health Service and Population Research, King’s College London Institute of Psychiatry Psychology and Neuroscience, London, UK; 4 Research and Community Services, Jigjiga University, Jijiga, Ethiopia; 5 King’s College London, London, UK; 6 Kabridahar University, Kebri Dehar, Ethiopia; 7 Maastricht University, Maastricht, The Netherlands; 8 Epidemiology and Biostatistics, Kabridahar University, Kebri Dehar, Ethiopia; 9 WHO, Addis Ababa, Ethiopia; 10 Ethiopia Ministry of Health, Jigjiga, Ethiopia; 11 Yeditepe Universitesi, Istanbul, Turkey; 12 Jigjiga University, Jijiga, Ethiopia; 13 Imperial College London, London, UK; 14 Addis Ababa University College of Health Sciences, Addis Ababa, Ethiopia; 15 University of Minnesota Medical School - Twin Cities Campus, Minneapolis, Minnesota, USA; 16 Dalarna University School of Health and Welfare, Falun, Sweden; 17 Department of Population Health Sciences, King’s College London, London, UK; 18 University of Edinburgh Division of Clinical and Surgical Sciences, Edinburgh, UK

**Keywords:** Amphetamine-Related Disorders, Community Mental Health Services, Mental Health, Psychiatry, Drug Misuse

## Abstract

**Background:**

Tackling the global burden of mental health conditions and substance misuse is an international priority. In recent years, strategic work has begun to develop mental health services in Ethiopia and strengthen human rights protection for people with mental health conditions. To inform mental health service planning in the Somali region of Ethiopia, the extent of mental health conditions and substance misuse requires quantification.

**Objective:**

We aimed to investigate associations between problematic *khat* use and mental health conditions in Ethiopia’s Somali Regional State (SRS).

**Method:**

We carried out a quantitative household survey of 844 randomly selected residences in two cities in Ethiopia’s SRS: the regional capital, Jigjiga (55.1%, n=465), and third largest city, Kabridahar (44.9%, n=379). We used the culturally validated Problematic Khat Use Screening Test and Mini International Neuropsychiatric Inventory to assess the prevalence and factors associated with mental health conditions and problematic *khat* use.

**Findings:**

Out of 844 participants, 18.6% (n=156) met criteria for major depression, 11.1% (n=92) met criteria for post-traumatic stress disorder (PTSD) and 5.7% (n=48) expressed suicidal ideation. The combined prevalence of these mental health conditions was 21.8% (n=186). Men reported more aggregated mental health conditions than women (30.6%, n=97 vs 16.9%, n=89). Adjusted regression analyses showed that people with problematic *khat* use had more than twice the prevalence of major depression (prevalence ratio (PR) 2.5, 95% CI 1.7 to 3.5), recurrent depression (PR 2.8, CI 1.8 to 4.4), suicidal ideation (PR 2.4 CI 1.9 to 5.3), PTSD (PR 3.1, CI 1.9 to 5.3) and any mental health condition (PR 2.4, CI 1.7 to 3.4) compared with people without problematic *khat* use.

**Conclusion:**

This study provides the first data regarding population mental health needs and harms associated with problematic *khat* use in the SRS, to inform the design, development and prioritisation of mental health services and substance regulation and policy.

**Clinical implications:**

Associations between problematic *khat* use and a range of poor mental health outcomes demonstrate the importance of targeted services for this population. Further research is needed to explore causal pathways underlying these associations.

**WHAT IS ALREADY KNOWN ON THIS TOPIC**

- Chewing the stimulant leaf, *khat,* is a popular means of self-medication in places where limited mental health services are provided. Many epidemiological studies report associations between *khat* consumption and both transient mental health symptoms and longer-lasting conditions, such as anger, anxiety, depression, psychosis and *khat* dependence.

**WHAT THIS STUDY ADDS**

- Although *khat* is usually used for social purposes, we found evidence of problematic use in the Somali Regional State. For the first time, we quantified associations between problematic *khat* use and mental health conditions such as major depression, post-traumatic stress disorder and suicidal ideation.

**HOW THIS STUDY MIGHT AFFECT RESEARCH, PRACTICE OR POLICY**

- This study identified associations between problematic *khat* use and mental health conditions. Addressing this dual burden requires multistakeholder recognition and response. Public health messaging and integrated clinical services are needed to address problematic *khat* use, mental health conditions and dual diagnoses. Research is needed to investigate underlying causal pathways and inform service development in this under-resourced East African setting.

## Introduction

 After several decades of recurrent conflict, the Somali Regional State (SRS) of Ethiopia has experienced a period of rebuilding and renewal since 2018.^1^ Nevertheless, protracted conflict and lack of mental health infrastructure have impacted population mental health, substance misuse (particularly *khat*) and livelihoods.^1^ National mental health priorities are variably implemented, in part due to lack of evidence-based data to support prioritisation by decision-makers.

*Khat* is commonly chewed for self-medication in places where mental health services are limited.^2^ *Khat* is a stimulant leaf, with psychoactive properties.^3 4^ The main active constituents of *khat*, cathinone and cathine, have amphetamine-like properties.^5^ Shortly after *khat* is chewed, these alkaloids are released and absorbed primarily through the oral mucosa and gastrointestinal tract.^6^ Khat chewing leads to an elevation of synaptic monoamines, especially in dopaminergic pathways, which underlies the typical acute effects such as euphoria, alertness, increased energy and reduced appetite.^6 7^ However, cathinone is chemically unstable and is rapidly metabolised into the less potent compounds of cathine and norephedrine.^8^ The rapid decline in cathinone levels, particularly in the context of stimulant rebound effects and development of tolerance with chronic use, is associated with a post-chewing ‘crash’.^8^ The crash is characterised by dysphoric mood, fatigue, irritability and other negative affective symptoms that may contribute to a broader pattern of mental distress.

Adverse health effects of *khat* have been extensively described.^9^ Epidemiological studies report associations between *khat* consumption and both transient mental health symptoms and longer lasting conditions, such as anxiety, depression, psychosis and *khat* dependence.^10 11^ *Khat* is also associated with physical health conditions including cardiovascular disease,^12^ hypertension,^13^ gastrointestinal disorders,^14^ dental erosion and malignant buccal lesions^15^ and chronic liver disease.^16^

Nonetheless, *khat* plays an important traditional cultural role. In the SRS, predominantly male chewers of *khat* engage in longer *khat-*chewing sessions to enter a dreamlike state known as the *mirqaan* (‘high’) stage.^17^ During this heightened mental state, men typically create grandiose plans for future opportunities and fantasise ideas. These experiences have been associated with poor mental health, especially when fantasies and *khat*-induced dreams do not materialise.^17^ *Mirqaan* is commonly followed by a ‘come down’ period when *khat*-fuelled dreams are recognised as being temporary. These euphoric dreams are typically considered to be transient delusions by clinicians, since they recede when the psychoactive effects of *khat* subside.^5^ The fluctuating mental state of *mirqaan* has been associated with major depression and psychotic episodes in a proportion of cases.^17^

Barriers to the development of a robust mental health system include lack of data to guide clinical investment and policy making. This study aimed to address this gap by understanding the associations between problematic *khat* use and mental health conditions.

### Objective

The objective of this study was to quantify the prevalence of common mental disorders and problematic *khat* use in Ethiopia’s SRS.

### Hypothesis

Adults with problematic *khat* use are more likely to experience common mental health conditions: (a) major depression and recurrent depressive disorder, (b) post-traumatic stress disorder (PTSD), (c) suicidal ideation and (d) any mental health condition than adults without problematic *khat* use.

## Methods

This study was a cross-sectional household survey conducted in two cities in the SRS: Jigjiga (the state capital) and Kabridahar (the third largest city). Ethiopia’s SRS has an estimated population of 6.7 million people, across 11 regional zones (counties) served by six administrative cities (Jigjiga, Kabridahar, Goday, Dhagahbuur, Kebribayah and Wajaale).

### Sample size

Sample size was estimated using Yamane’s method^18^: n=*N*/(1+*N*e²), where *N* is the total population size and *e* is the acceptable margin of error (set at 5%). For Jigjiga and surrounding urban and rural areas (n=1,256,567), this yielded a required sample of n=1 256 567/(1+1 256 567 × 0.05²) = 400. For Kabridahar and its surrounding urban and rural areas (n=4 04 280), the required sample was n=4 04 280/(1+4 04 280 × 0.05²) = 399.

### Sampling strategy

We numbered the 22 districts of Jigjiga city and the 10 districts of Kabridahar, each in alphabetical order. We used a lottery method to select 10 districts out of 22 in Jigjiga and six out of 10 in Kabridahar.

In each selected district, a cross-sectional survey was administered by trained, supervised research assistants. Research assistants approached randomly selected households in each district to invite adults aged 18 years or older to take part. Since there are no street names or household door numbers in either city, research staff sampled every third house in a given street within randomly selected districts.

After initial screening for inclusion and exclusion criteria, staff provided eligible household members with an information sheet and verbal explanation about the study and invited them to give informed consent. The head of the household or any adult above the age of 18 years was interviewed from each house.

For Jigjiga city, we used a reduced sampling strategy to select 47 surveys in each of 10 randomly selected districts: n=470 surveys. Excluded: five questionnaires which did not meet inclusion criteria or were incomplete. Final sample from Jigjiga: n=465. For Kabridahar city, we used an increased sampling strategy to select 66 surveys in each of the six randomly selected districts of Kabridahar: n=396 surveys. Excluded questionnaires: 17. Final sample from Kabridahar: n=379. In total, 866 participants completed the survey, of whom data from 844 were included in the final analysis.

### Participants, study population and research processes

The target population was adults aged 18 years or older who lived in the SRS. The source population was all adults living in two cities in the SRS. The study population was a random sample of adult households who met the inclusion criteria.

Inclusion criteria for participation were adults aged 18 years and older, who provided informed consent and felt well enough to take part. Exclusion criteria were individuals younger than 18 years old and adults who were unable to understand the information provided or to verbally respond to questions asked in Somali or English. Participants who reported or displayed signs of confusion and acute physical health symptoms were not eligible to take part and were supported to access the local health service.

### Maximising response rate

Traditionally, this is a young population setting with adult participants always present at any given house to look after young children. In addition, and in order to maximise response rates, we combined various data collection times from weekday to weekends and from morning sessions to afternoon ones. Where a household had no adult present, researchers were advised to recruit from the next eligible household participants. All these efforts significantly improved our response rate. Of the 866 eligible households, all participated but due to incomplete data, the analyses are based on responses from 844 participants (97.4%).

### Study measures

The survey comprised three subsections. First, a demographic questionnaire asked about age, gender, marital status, education, employment and religion. Second, we used the Problematic Khat Use Screening Test (PKUST-17).^19^ We assessed the prevalence and nature of *khat* use. Mihretu and colleagues^19^ developed a culturally valid measure to differentiate non-harmful *khat* use from problematic use in the Ethiopian context, the PKUST-17.^19^ The PKUST-17^19^ has 17 items regarding negative experiences of *khat* use in the past 3 months. The scale defines problematic *khat* use in terms of addictive patterns of use, such as using *khat* often and always to treat withdrawal symptoms. ‘Unproblematic’ use includes never (or rarely) experiencing stress, reduced motivation, irritability or depressive symptoms when unable to obtain *khat*. We operationalised problematic khat use as a score >17.

Third, we used the Mini-International Neuropsychiatric Inventory (MINI) questionnaire, which has been translated and validated in Somali^20^ and has good validity and reliability.^21 22^ The MINI is a structured instrument suitable for delivery by trained lay and non-clinical interviewers.^22^ The MINI derives from WHO’s International Classification of Diseases-10 and from the DSM-IV (Diagnostic and Statistics Manual of Mental Disorder IV-) compatible mental health diagnoses (see online supplemental appendix 1). For this study, we measured common mental health conditions or symptoms captured by the MINI: current major depression and recurrent major depression, PTSD, suicidal ideation and all mental health conditions.

### Managing Risks

When asked a general question about how they felt, any prospective participants disclosing suicidal thoughts or plans were supported by the research assistant, who followed a study protocol for responding to mental health risks. This entailed listening and gaining further information. Those for whom mental health risks, such as suicidal ideation, were identified were supported to access the nearest hospital emergency department, either voluntarily or with the support of the research assistant. Further information about where to obtain treatment for mental health conditions in Jigjiga and Kabridahar was provided as needed. Participants were interviewed in the most private location within the household, for example, *daarad* (the veranda). Participants were informed that they could stop the interview at any time or take a break. Participants did not receive compensation or incentives to take part.

### Analysis

We used SPSS (V.25) and Stata (V.18) to perform descriptive analyses of sociodemographic characteristics, mental health conditions and problematic *khat* use, stratified by gender. Throughout, we used complete case analyses. We then carried out hypothesis-driven analyses of the association between problematic *khat* use (PKUST total score >17) as the independent variable and mental health conditions as the dependent variable (separate models for current depression, recurrent depression, suicidality, PTSD and a composite of ‘any mental health condition’) adjusting for potential confounders of age, gender, educational level and marital status. Prevalence ratio was chosen instead of OR as the appropriate measure of effect as they are less liable to misinterpretation.^23^ Prevalence ratios were estimated using a Poisson working model and sandwich estimators of the standard errors.^24^ We carried out further analyses on sociodemographic characteristics, problematic *khat* use and mental health conditions stratified by city of residence.

## Findings

### Sociodemographic characteristics

We analysed responses from 844 eligible household participants. Table 1 summarises participants’ demographic characteristics. Of the 844 participants, 465 (55.1%) were recruited in Jigjiga and 379 (44.9%) in Kabridahar. Over half of the participants (n=526, 62.3%) were female and the remainder, male (n=318, 37.7%). Most participants were Muslim (n=804, 95.3%) and the rest, mostly, Christian. The majority of participants was unemployed (n=546, 64.7% vs n=285, 33.8%). Half of the participants had no formal education (n=418, 49.5%). Most participants (n=526, 62.3%) were married, 177 (21%) had never married and 65 (7.7%) were divorced. The largest proportions of participants were aged between 21 years and 29 years and 30 years and 39 years (n=225, 26.7% and n=233, 27.6%, respectively). The smallest proportion of participants was aged 70 years and older (n=15, 1.8%). See table 1 for more details.

**Table 1:** Sociodemographic characteristics

Characteristic	Categories	n (%)
Location	Jigjiga	465 (55.1)
	Kebridahar	379 (44.9)
Gender	Male	318 (37.7)
	Female	526 (62.3)
Age category (n=836)	18–20 years	95 (11.4)
	20–29 years	225 (26.9)
	30–39 years	233 (27.9)
	40–49 years	158 (18.9)
	50 years and above	125 (15.0)
Educational level (n=835)	No formal education	418 (50.1)
	Primary/intermediate	175 (21.0)
	Secondary	108 (12.9)
	Post-secondary	134 (16.1)
Marital status (n=836)	Married	526 (62.9)
	Never married	177 (21.2)
	Divorced or separated	89 (10.7)
	Widowed	44 (5.3)
Religion (n=827)	Muslim	804 (97.2)
	Others	23 (2.7)
Employment status (n=831)	Employed	285 (34.3)
	Unemployed	546 (65.7)
Employment type (n=830)	Housework (non-salaried)	389 (46.9)
	Civil servant	121 (14.6)
	Merchant/trader	68 (8.2)
	Farmer	25 (3.0)
	Student	61 (7.4)
	Unemployed	69 (8.3)
	Labourer	64 (7.7)
	Other	33 (4.0)

### Prevalence of mental health conditions

Table 2 summarises the prevalence of mental health conditions using the MINI (n=844).^21 22^

**Table 2:** Prevalence of mental health conditions (Mini International Diagnostic Inventory) (N=844)

Mental health condition	Prevalence in men n (%)	Prevalence in women n (%)	Overall prevalence n (%)
Current major depression (n=841)	89 (28.1)	67 (12.8)	156 (18.6)
Recurrent major depression (n=837)	72 (22.7)	31 (6.0)	103 (12.3)
Moderate/high risk suicidality (n=843)	29 (9.2)	19 (3.6)	48 (5.7)
Post-traumatic stress disorder (n=831)	52 (16.8)	40 (7.7)	92 (11.1)
Any mental health condition (n=820)	97 (30.6)	89 (16.9)	179 (21.8)

The prevalence of current major depressive disorder was 18.6% (n=156) and the prevalence of recurrent depressive disorder was 12.3% (n=103). The prevalence of PTSD was 10.9% (n=92) and the prevalence of suicidal ideation was 5.7% (n=48). The prevalence of any mental health condition (major or recurrent depressive disorder, PTSD or suicidal ideation) was 21.8% (n=186).

There were interesting differences in mental health conditions by gender. The prevalence rates of major depression, recurrent major depression, PTSD and suicidal ideation were higher in men than women. Because of the role of *khat* in mental illness in East Africa, men are usually expected to experience more mental health conditions than women. On the other hand, depression is widely known to be higher in women than men. However, we did not find the usual excess of depression in women from our survey. See discussion for further elaborations.

### Prevalence of problematic *khat* use

Table 3 summarises item frequency of PKUST-17 scores (n=844).^19^

**Table 3:** Item frequency of the Problematic *Khat* Use Tool (PKUST-17) (N=844)

	PKUST-17 item	Response category	N (%)
1	During the past 3 months, how often did you chew *khat*? (n=834)	None/less than once in 3 months	699 (83.8)
		1–3 days per month	32 (3.8)
		1 day or 2 days in a week	32 (3.8)
		3 days or 4 days in a week	24 (2.9)
		Daily or almost daily	47 (5.6)
2	During the past 3 months, when you chewed *khat*, on average, how much time did you spend chewing without engaging in important tasks? (n=836)	None/an hour or less	697 (83.4)
		2–3 hours	35 (4.2)
		5 hours	32 (3.8)
		5–6 hours	26 (3.1)
		6 hours or more	46 (5.5)
3	In the past 3 months, how often do you chew *khat* to treat distressing experiences or depression when the *khat* is withdrawn? (n=841)	Never	701 (83.4)
		Rarely	25 (3.0)
		Sometimes	39 (4.6)
		Quite often	37 (4.4)
		Almost always	39 (4.6)
4	In the past 3 months, how often do you experience craving for *khat*? (n=836)	Never	694 (83)
		Rarely	22 (2.6)
		Sometimes	31 (3.7)
		Quite often	43 (5.1)
		Almost always	46 (5.5)
5	In the past 3 months, how often do you experience distressing emotional or behaviour symptoms when the *khat* is withdrawn? (n=840)	None/never	694 (82.6)
		Rarely	29 (3.5)
		Sometimes	36 (4.3)
		Quite often	32 (3.8)
		Almost always	49 (5.8)
6	During the past 3 months, how much has *khat* led to financial problems? (n=839)	None/never	698 (83.2)
		Rarely	22 (2.6)
		Sometimes	23 (2.7)
		Quite often	37 (4.4)
		Almost always	59 (7)
7	In the past 3 months, to what extent have you failed to do what was expected of you at work because of chewing *khat*? (n=840)	None/never	707 (84.2)
		Rarely	22 (2.6)
		Sometimes	33 (3.9)
		Quite often	41 (4.9)
		Almost always	37 (4.4)
8	During the past 3 months, how often do you feel depressed when you did not chew *khat*? (n=834)	Never	718 (85.7)
		Rarely	21 (2.5)
		Sometimes	37 (4.4)
		Quite often	24 (2.9)
		Almost always	37 (4.4)
9	During the past 3 months, how often do you experience irritability when you did not chew *khat*? (n=837)	Never	712 (85.1)
		Rarely	39 (4.7)
		Sometimes	21 (2.5)
		Quite often	29 (3.5)
		Almost always	36 (4.3)
10	During the past 3 months, how often do you experience vivid, unpleasant dreams when you did not chew *khat*? (n=840)	Never	717 (85.3)
		Rarely	28 (3.3)
		Sometimes	34 (4.0)
		Quite often	27 (3.2)
		Almost always	34 (4.0)
11	During the past 3 months, how often do you experience tear falling that blurred your vision when you did not chew *khat*? (n=840)	Never	717 (85.4)
		Rarely	26 (3.1)
		Sometimes	38 (4.5)
		Quite often	30 (3.6)
		Almost always	29 (3.5)
12	During the past 3 months, how often do you experience frequent yawning when you did not chew *khat*? (n=841)	Never	710 (84.4)
		Rarely	33 (3.9)
		Sometimes	32 (3.8)
		Quite often	34 (4.0)
		Almost always	32 (3.8)
13	During the past 3 months, how often has your work motivation reduced when you did not chew *khat*? (n=837)	Never	717 (85.6)
		Rarely	24 (2.9)
		Sometimes	26 (3.1)
		Quite often	37 (4.4)
		Almost always	33 (3.9)
14	During the past 3 months, how often do you experience serious fatigue or reduced energy when you did not chew *khat*? (n=840)	Never	714 (85)
		Rarely	34 (4.0)
		Sometimes	32 (3.8)
		Quite often	32 (3.8)
		Almost always	28 (3.3)
15	During the past 3 months, to what extent have you increased your amount of *khat* to get the desired effect (n=840)	Never	706 (84.1)
		Rarely	28 (3.3)
		Sometimes	38 (4.5)
		Quite often	38 (4.5)
		Almost always	30 (3.6)
16	During the past 3 months, how often do you experience restlessness when you did not chew *khat*? (n=840)	Never	714 (85)
		Rarely	34 (4.0)
		Sometimes	33 (3.9)
		Quite often	27 (3.2)
		Almost always	32 (3.8)
17	During the past 3 months, how often do you experience a marked diminished effect with continued use of the same amount of *khat*? (n=841)	Never	706 (5.7)
		Rarely	35 (4.2)
		Sometimes	31 (3.7)
		Quite often	39 (4.6)
		Almost always	30 (3.6)

We identified problematic *khat* use in 113 participants (13.9%). There were gender differences, with 106 men (36.1%) and seven women (1.4%) reporting problematic use. Respondents reported a range of withdrawal symptoms and adverse impacts of *khat* chewing, with 89 participants (10.6%) experiencing craving quite often or almost always when not chewing *khat*. Distressing emotional or behavioural symptoms were experienced by 49 people (5.8%) when *khat* was withdrawn. Financial problems due to *khat* were the most common adverse impact, reported by participants (11.4%) in the categories of quite often or almost always.

### Regression analysis of problematic *khat* use and mental health conditions

Table 4 summarises adjusted regression analysis of problematic *khat* use and mental health conditions.

**Table 4:** Adjusted* regression analysis of problematic *khat* use and mental health conditions

Dependent variable	Independent variable		Crude prevalence ratio (95% CIs)	Adjusted prevalence ratio (95% CIs)
	Problematic *Khat* use (>17 on PKUST) n (%)	Not problematic *khat* use (<18 on PKUST) n (%)		
Current depression (n=808)	52 (46.4)	92 (13.2)	3.5 (2.7 to 4.6)	2.5 (1.7 to 3.5) (n=785)
Recurrent depression (n=804)	43 (38.4)	52 (7.5)	5.1 (3.6 to 7.3)	2.8 (1.8 to 4.4) (n=781)
Moderate/high risk suicidality (n=810)	17 (15.2)	27 (3.9)	3.9 (2.2 to 7.0)	2.4 (1.2 to 4.8) (n=787)
Post-traumatic stress disorder (n=800)	33 (30.0)	53 (7.7)	3.9 (2.7 to 5.7)	3.1 (1.9 to 5.3) (n=777)
Any mental health condition (n=789)	53 (49.5)	112 (16.4)	3.0 (2.3 to 3.9)	2.4 (1.7 to 3.4) (n=766)

* Adjusted for gender, age category, educational level and marital status.

PKUST, Problematic Khat Use Tool.

The prevalence of major depressive disorder, recurrent depressive disorder, suicidal ideation or PTSD was 2.5–3.1 times higher in people reporting problematic *khat* use, after adjusting for age, gender, educational level and marital status. Nearly half (49.5%) of the participants with problematic *khat* use had one or more mental health conditions.

### Differences between Jigjiga and Kabridahar

Stratified findings by location (Jigjiga and Kabridahar) are presented in online supplemental tables 1–3. The proportion of female participants was lower in Jigjiga compared with Kabridahar (58.7% vs 66.8%). The Jigjiga sample also had an older age distribution, a lower percentage of divorced, separated or widowed participants and lower levels of unemployment (56.6% vs 73.3%). There was no significant difference in the prevalence of major depression, PTSD or problematic *khat* use between locations, but the prevalence of suicidal ideation was significantly lower in Jigjiga (4.1% vs 7.7%) than in Kabridahar. Prevalence ratios for problematic *khat* use and mental health conditions did not differ significantly across locations except for suicidal ideation (Z −2.33 p=0.020), although absolute numbers were small.

## Discussion

This household survey provides new data on associations between problematic *khat* use and mental health conditions after adjusting for age, gender, education and marital status in Ethiopia’s SRS. Compared with those whose use of *khat* was ‘non-problematic’ (scoring low for features of dependence), almost half of the participants with problematic *khat* use (scoring high for features of dependence) met criteria for major depressive disorder, PTSD or suicidal ideation. This finding demonstrates distinct mental health profiles between groups of adults with different *khat* use patterns.

Emerging from regional war and conflict, and from four decades of marginalisation within Ethiopia, we found that the prevalence of mental health conditions in the SRS was similar to that in the neighbouring Hararghe region.^25^ The prevalence of PTSD was similar to that of Somali immigrant samples^26^ but much lower than that in refugee samples.^27^ Strong religious and sociocultural practices may have contributed to relatively low levels of PTSD, as this was the case with the previous Somali studies.^28^

Widespread *khat* consumption in the SRS is socially condoned and strongly associated with social interactions which may have mental health benefits for those without dependent or ‘problematic’ use. In contrast to the relatively low PTSD prevalence across the sample, almost one in three participants with problematic *khat* use met criteria for PTSD. This finding may indicate that *khat* is chewed by some for self-medication in this post-conflict setting with limited mental health service provision.^2^

The prevalence of problematic *khat* use was higher among male compared with female participants. *Khat* is traditionally chewed by men in large groups in private spaces to foster a climate of social occasion and enjoyment.^17^ Given that comparable data are not available for pre-conflict or intra-conflict associations between *khat* use and mental health conditions in the SRS, it is not possible to be certain whether increasing numbers of citizens are chewing *khat* to cope with mental health symptoms. Longitudinal designs would provide valuable data to track evolving patterns of *khat* use and associated harms. Our findings support the potential for a cyclical relationship in a subgroup of people, whereby *khat* is initially chewed to cope with distressing emotions but then develops into dependent and problematic use.

Our recent qualitative study explored community perceptions of mental health, stigma of mental illness and service utilisation in the SRS.^29^ We found that most participants had a binary conception of mental ill-health: one is either mad/crazy (*waali*) or not. Most participants attributed mental health conditions to spiritual causes and predominantly advocated seeking help from traditional providers.^29^ Society was particularly blamed for stigmatising people with mental health conditions. In this context of unmet mental health needs and low service provision,^1^ self-medication with *khat* may offer a culturally sanctioned means of evading stigma.^29^

Culturally rooted stigma of mental illness may also explain why more women reported less major depression than men in our survey. Because of the severity of stigma of mental illness in the SRS,^29^ more women might have disclosed less depression and depressive symptoms than men. Our sample was disproportionately male in Jigjiga, it is expected that the capital city attracts more male migrants seeking employment, business and education opportunities. Divorce, separation and widowhood were more prevalent in Kabridahar than Jigjiga. Long-term insecurity, displacement and instability experienced by the community^1^ may have impacted relationships adversely.

Our findings establish the extent of population mental health conditions and substance misuse needs in Ethiopia’s SRS. Public information around physical and mental health harms of *khat*, regulation of *khat* sale and provision of addiction services to address dependent use of *khat* is needed in addition to mental health services. In Muslim countries, where *khat* is more commonly chewed than alcohol consumed, research is needed to adapt evidence-based interventions for the cultural and substance use context, before evaluating feasibility of implementation and, ultimately, efficacy for reducing harmful and dependent use. While we did not measure intimate partner violence perpetration in this study, it is often associated with substance misuse, including *khat* chewing in Ethiopia. Future research should measure other social harms associated with substance misuse in other settings to inform public health strategies for addressing *khat* as well as mental ill-health in the SRS.

Developing the region’s first mental health system is an ambitious goal requiring time and resources beyond those currently available, in times of exceptionally restricted funding for global health.^30^ Regional states and federal governments should first prioritise embedding mental health within primary care, as recommended by WHO, with a focus on developing the communication skills required for sensitive mental health conversations in the healthcare workforce in low-resourced settings such as the SRS.

### Strengths and limitations

This study is the first household survey of mental health and *khat* use in the SRS of Ethiopia. For the first time, we collected comprehensive data to inform the development of a mental health system in the SRS, highlighting common mental disorders and problematic *khat* use as priorities requiring intervention. Our survey responds to prior calls to strengthen mental health leadership and build mental health research capacity in resource-limited Global South settings, contributing evidence towards establishing a formal SRS mental health system. The findings of this study will promote research leadership in the SRS to work in partnership with policy makers to address care needs for prioritised conditions (substance misuse, suicidal ideation, major depression, PTSD and psychosis).

A further strength is the collaborative involvement in this study of key partners for responding to the findings, including the Ethiopian Ministry of Health, Somali Region Health Bureau, WHO Ethiopia, local and international universities and organisations. Nevertheless, our sampling strategy was limited by the fact that in SRS, few streets have names and houses are not numbered. Due to the layout of streets in Jigjiga and Kabridahar, our random sampling strategy collected data from the streets with the most concentrated numbers of households. This may have introduced bias into our sample, since families living in these more populous streets and areas could have differed systematically. Likewise, our sample was restricted by those members of the household who were at home when researchers visited and agreed to take part.

In this population affected by long periods of armed conflict, recall bias may have influenced disclosure of mental health symptoms. This possibility is mitigated by the fact that our prevalence estimates are consistent with those we found in similar Somali populations. Although this is the first survey of the prevalence and factors associated with mental health conditions in the SRS, the findings cannot be generalised beyond the two zones and cities where the data are collected. Given very limited prior research in the SRS, further research is required to investigate patterns and predictors of use and mental health conditions across the region.

Moreover, we have given priority to the participants’ welfare and have given them the choice to not answer any questions they did not feel comfortable with answering. For example, although 152 participants disclosed *khat* chewing habits, not everyone answered subsequent questions. Therefore, given the impact of stigma on people with mental health conditions in the SRS, and since the adverse effects of problematic *khat* are linked to a decline into mental illness, we cannot rule out diminished disclosure in our survey, leading us to underestimate the true prevalence of *khat* use, mental health conditions and suicidal ideation.

## Conclusions

Community mental health of regional states in low-income countries is markedly under-researched. In post-conflict settings, socially condoned *khat* use places a subgroup of people at risk of problematic use, which is associated with elevated risk of mental health conditions and suicidal ideation.

Improving mental health services and enhancing human rights protections for people with mental health conditions are priorities in Ethiopia. In addition to addressing severe gaps in mental health service provision, studies such as ours demonstrate increased prioritisation of mental health research in low-resource settings in Africa. Like our survey, other studies in Ethiopia did not also find the expected excess of depression in women, for example, Hailemarian’s 2012 National Health Survey in Ethiopia. We recommend paying extra attention to gender differences in mental health in East Africa, carrying out more appropriate studies with longitudinal designs.

Tackling problematic *khat* use and associated mental health conditions requires multistakeholder collaborations between researchers, clinicians, community partners, Ministries of Health, regional health bureau, service user organisations and local, national and international agencies.

## Supplementary material

10.1136/bmjment-2025-302388 online supplemental file 1

## Data Availability

Data are available upon reasonable request.
