# Supplementary material for: Impact of problematic Khat use on mental health in Ethiopia’s Somali Regional State: a population study
Source: BMJ Ment Health. 2026 Jul 28;29(1):e302388. doi: 10.1136/bmjment-2025-302388 (PMC13423073; doi:10.1136/bmjment-2025-302388)
Supplement: online supplemental file 1 [file bmjment-29-1-s001.docx]

**Supplementary Files**

**Impact of problematic Khat use on mental health in Ethiopia’s Somali Regional State**

**A population study**

**Supplementary Table 1:** **Sociodemographic characteristics stratified by city of residence**

| **Characteristic** | **Categories** | **Jigjiga**  **n (%)** | **Kabridahar**  **n (%)** | **Overall**  **n (%)** |
| --- | --- | --- | --- | --- |
| Gender | Male | 192 (41.3) | 126 (33.3) | X2(1) 5.7552;  p = 0.016 |
|  | Female | 273 (58.7) | 253 (66.8) |  |
| Age category  (n=836) | 18-20 years | 28 (7.4) | 67 (14.6) | Z = -4.38  p < 0.001 |
|  | 20-29 years | 83 (22.0) | 142 (31.0) |  |
|  | 30-39 years | 118 (31.2) | 115 (25.1) |  |
|  | 40-49 years | 84 (22.2) | 74 (16.2) |  |
|  | 50 years and above | 65 (7.2) | 60 (13.1) |  |
| Educational level  (n=835) | No formal education | 177 (47.3) | 241 (52.3) | Z=1.18  p = 0.236 |
|  | Primary/intermediate | 104 (27.8) | 71 (15.4) |  |
|  | Secondary | 47 (12.6) | 61 (13.2) |  |
|  | Post-secondary | 46 (12.3) | 88 (19.1) |  |
| Marital status  (n=836) | Married | 283 (61.9) | 243 (64.1) | X2(3) 60.9348  p < 0.001 |
|  | Never married | 133 (29.1) | 44 (11.6) |  |
|  | Divorced or separated | 29 (6.4) | 60 (15.8) |  |
|  | Widowed | 12 (2.6) | 32 (8.4) |  |
| Religion | Muslim | 365 (98.4) | 439 (96.3) | X2(1) 3.3710;  p = 0.066 |
|  | Others | 6 (1.6) | 17 (3.7) |  |
| Employment status  (n=831) | Unemployed | 214 (56.6) | 332 (73.3) | X2(1) 25.4277;  p < 0.001 |

**Supplementary Table 2:** **Mental health conditions and problematic khat use stratified by city of residence**

| **Mental health or substance use condition** | **Jigjiga**  **n (%)** | **Kabridahar**  **n (%)** | **Pearson X2 (degrees of freedom); p-value** |
| --- | --- | --- | --- |
| Current major depression | 87 (18.8) | 69 (18.3) | 0.0396(1);  p=0.842 |
| Recurrent major depression | 63 (13.6) | 40 (10.8) | 1.4968(1);  p=0.221 |
| Post-traumatic stress disorder | 58 (12.5) | 24 (9.6) | 2.1792(1);  p=0.1420 |
| **Moderate/high suicide risk** | **19 (4.1)** | **29 (7.7)** | **4.9149(1);**  **p=0.027** |
| Any common mental health condition | 97 (20.9) | 89 (23.5) | 0.8740(1);  p=0.350 |
| Problematic khat use | 57 (12.9) | 56 (15.2) | 0.8719(1);  P=0.350 |

**Supplementary Table 3: Prevalence ratio for problematic khat use by mental health conditions**

|  | **Prevalence ratio for problematic khat use by mental health conditions** | |
| --- | --- | --- |
| **Mental health condition** | **Interaction with location (Jigjiga vs Kabridahar)** | |
|  | In crude analysis | In adjusted analysis* |
| Current major depression | NS | NS |
| Recurrent major depression | NS | NS |
| Post-traumatic stress disorder | NS | NS |
| Moderate/high suicide risk | Z-2.24  p = 0.025 | Z -2.33  p = 0.020 |
| Any common mental health condition | NS | NS |

NS = Non-significant (p<0.05)

*Adjusted for age, gender, education and marital status

Appendix 1

**MODULES** **TIME FRAME CRITERIA DSM-IV ICD-10**

A MAJOR DEPRESSIVE EPISODE Current (2 weeks) 🞏 296.20-296.26 Single F32.x

Recurrent 🞏 296.30-296.36 Recurrent F33.x

MDE WITH MELANCHOLIC FEATURES Current (2 weeks) 🞏 296.20-296.26 Single F32.x

Optional 296.30-296.36 Recurrent F33.x

B DYSTHYMIA Current (Past 2 years) 🞏 300.4 F34.1

C SUICIDALITY Current (Past Month) 🞏

Risk: 🞏 Low 🞏 Medium 🞏 High

D MANIC EPISODE Current 🞏 296.00-296.06 F30.x-F31.9

Past 🞏

HYPOMANIC EPISODE Current 🞏 296.80-296.89F31.8- F31.9/F34.0

Past 🞏

E PANIC DISORDER Current (Past Month) 🞏 300.01/300.21 F40.01-F41.0

Lifetime 🞏

F AGORAPHOBIA Current 🞏 300.22 F40.00

G SOCIAL PHOBIA (Social Anxiety Disorder) Current (Past Month) 🞏 300.23 F40.1

H OBSESSIVE-COMPULSIVE DISORDER Current (Past Month) 🞏 300.3 F42.8

I POSTTRAUMATIC STRESS DISORDER Current (Past Month) 🞏 309.81 F43.1

Optional

J ALCOHOL DEPENDENCE Past 12 Months 🞏 303.9 F10.2x

ALCOHOL ABUSE Past 12 Months 🞏 305.00 F10.1

K SUBSTANCE DEPENDENCE (Non-alcohol) Past 12 Months 🞏 304.00-.90/305.20-.90 F11.1-F19.1

SUBSTANCE ABUSE (Non-alcohol) Past 12 Months 🞏 304.00-.90/305.20-.90 F11.1-F19.1

L PSYCHOTIC DISORDERS Lifetime 🞏 295.10-295.90/297.1/ F20.xx-F29

Current 🞏 297.3/293.81/293.82/

293.89/298.8/298.9

MOOD DISORDER WITH PSYCHOTIC FEATURESCurrent 🞏 296.24 F32.3/F33.3

M ANOREXIA NERVOSA Current (Past 3 Months) 🞏 307.1 F50.0

N BULIMIA NERVOSA Current (Past 3 Months) 🞏 307.51 F50.2

ANOREXIA NERVOSA, BINGE EATING/PURGING TYPECurrent 🞏 307.1 F50.0

O GENERALIZED ANXIETY DISORDER Current (Past 6 Months) 🞏 300.02 F41.1

P ANTISOCIAL PERSONALITY DISORDER Lifetime 🞏 301.7 F60.2

Optional
